# Whole transcriptome analysis reveals changes in expression of immune-related genes during and after bleaching in a reef-building coral

**DOI:** 10.1098/rsos.140214

**Published:** 2015-04-01

**Authors:** Jorge H. Pinzón, Bishoy Kamel, Colleen A. Burge, C. Drew Harvell, Mónica Medina, Ernesto Weil, Laura D. Mydlarz

**Affiliations:** 1 Department of Biology, University of Texas Arlington, Arlington, TX 76016, USA; 2 Department of Biology, The Pennsylvania State University, State College, PA 16802, USA; 3 Institute of Marine and Environmental Technology, University of Maryland Baltimore County Columbus Center, 701 East Pratt Street, Baltimore, MD 21202, USA; 4 Department of Ecology and Evolutionary Biology, Cornell University, Ithaca, NY 14853, USA; 5 Department of Marine Sciences, University of Puerto Rico—Mayagüez, La Parguera, PR 00865, USA

**Keywords:** climate change, thermal stress, coral bleaching, symbiosis dissociation, transcriptomics, immune system

## Abstract

Climate change is negatively affecting the stability of natural ecosystems, especially coral reefs. The dissociation of the symbiosis between reef-building corals and their algal symbiont, or coral bleaching, has been linked to increased sea surface temperatures. Coral bleaching has significant impacts on corals, including an increase in disease outbreaks that can permanently change the entire reef ecosystem. Yet, little is known about the impacts of coral bleaching on the coral immune system. In this study, whole transcriptome analysis of the coral holobiont and each of the associate components (i.e. coral host, algal symbiont and other associated microorganisms) was used to determine changes in gene expression in corals affected by a natural bleaching event as well as during the recovery phase. The main findings include evidence that the coral holobiont and the coral host have different responses to bleaching, and the host immune system appears suppressed even a year after a bleaching event. These results support the hypothesis that coral bleaching changes the expression of innate immune genes of corals, and these effects can last even after recovery of symbiont populations. Research on the role of immunity on coral's resistance to stressors can help make informed predictions on the future of corals and coral reefs.

## 2. Introduction

Environmental changes associated with climate change are affecting natural ecosystems [1–3]. Stressors, such as elevated sea surface temperature (i.e. thermal stress) and ocean acidification, are major causes of the decline of coral populations and deterioration of coral reefs [4–8]. Thermal stress has been associated with coral bleaching (i.e. disruption of the coral–algae symbiosis) [9], one of the most serious threats to coral health [10]. Coral bleaching affects the reproduction [11], growth, development [12] and health [13] of corals and weakens the structure and functionality of the reefs [14], ultimately affecting other reef inhabitants [6]. Coral bleaching events have become more frequent and devastating in the last several decades [15–17].

In a symbiotic relationship, the survival of both partners depends on their individual physiological capabilities and their combined resilience and resistance. In corals, the dissociation of the coral–algae symbiosis is associated with an increase in temperature of only a few degrees over a prolonged period of time [9]. During bleaching, the host tissue loses its symbiont cells, giving colonies a white appearance [18–20]. Depending on the intensity and duration of the stress, coral colonies can either recover normal symbiont densities and gain back typical coloration, or lose tissue, or die. Aside from colony mortality or tissue necrosis, other immediate effects of coral bleaching include: cessation of skeletal growth [21]; reduction in epithelial tissue thickness [22], larval survival [12,23] and protein synthesis [24]; appearance of diseases [25]; and increase in disease-related mortality [26]. Some of the effects can extend years after bleaching with bleached colonies showing a reduction in tissue biomass, as well as in protein and lipid content [27]. Bleached corals can also halt or delay the onset of oogenesis [11] and show an increase in disease prevalence compared with corals that did not bleach [28,29].

Evidence suggests that as coral bleaching become more common, so do disease outbreaks [28]. Diseases, such as white plague and yellow band disease in *Orbicella faveolata*, dark spots in *Siderastraea siderea* and black band in *Colpophyllia natans*, occur after bleaching events [28,29]. Additionally, coral bleaching can initiate the appearance of new infections (white plague in *O. faveolata*), or an increase in disease severity in diseases that were already present (yellow band and white plague in *Orbicella* spp. [28,29]). The relationship between coral bleaching and disease outbreaks suggests that the host's innate immune system is affected by bleaching and the changes persist long after the stressful conditions are over [30].

It has been suggested that within a species, corals living in naturally warm environments have an increased tolerance to temperature stress compared with colonies inhabiting cooler environments [31–34]. Evidence from transcriptomic analyses has shown that colonies exposed to high temperatures, for relatively low periods of time, have the capacity to increase expression of temperature-tolerant genes during thermal stress [31]. Additionally, under experimental conditions, two Caribbean corals (*Acropora palmata* and *O. faveolata*) appear to have similar responses in gene expression during thermal stress [35,36]. Responses of these species to increased temperatures include: increases in heat shock and antioxidant gene expression; decrease in expression of calcium homeostasis and ribosomal proteins; restructuring of the extracellular matrix; and rearrangement of the actin cytoskeleton [35,36]. However, the impacts of natural thermally induced bleaching on a coral's cellular and molecular machinery remain largely unknown.

**Figure 1.**
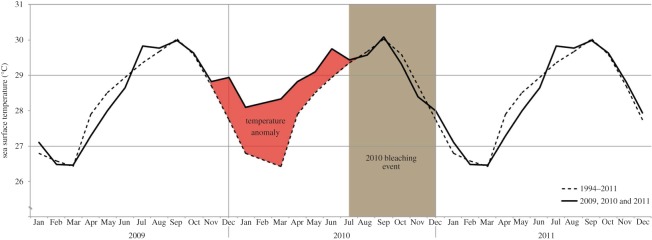
Comparison of the monthly average sea surface temperatures from 1994 to 2011 (dashed line) and the monthly average temperature observed in 2009, 2010 and 2011 (continuous line). In La Parguera, the 2010 bleaching event (brown box) lasted from June–July to November–December and is linked to the continuous temperature anomaly (red box) observed between November 2009 and June–July 2010. During the temperature stress period, temperatures were 1 to 2°C higher than the average for the region.

Although genomic and transcriptomic resources have become common tools to study coral responses and tolerance to environmental changes, impacts of these events on the coral immune system remain largely understudied [30,37,38]. The purpose of this study was to assess the effects of a natural bleaching event on genes involved in the innate immune system of the Caribbean coral *O. faveolata*. In 2010, corals and coral reefs around the world experienced thermal stress that resulted in widespread coral bleaching [39–41]. In the Caribbean, reefs off the Puerto Rican coastline were no exception. In La Parguera (southwest Puerto Rico), reefs experienced elevated temperatures (up to 2°C above average) from November 2009 through June/July 2010 (figure 1). Following this prolonged temperature anomaly, colonies from many coral species bleached and remained bleached through November/December 2010. Approximately 40% of *O. faveolata* colonies bleached (E. Weil, unpublished data). During this bleaching event, bleached and unbleached *O. faveolata* colonies were tagged and followed for 11 months. Metatranscriptome analyses (RNA-seq) were performed on tissue samples collected during the bleaching (November 2010), and at two time periods after the bleaching (March and October 2011). Results from the comparisons between bleached and unbleached colonies support the hypothesis that coral bleaching, due to thermal stress, affects the expression of innate immune genes of corals, and these effects can last at least 1 year after the event.

## 3. Material and methods

### 3.1 Tissues collection

**Figure 2.**
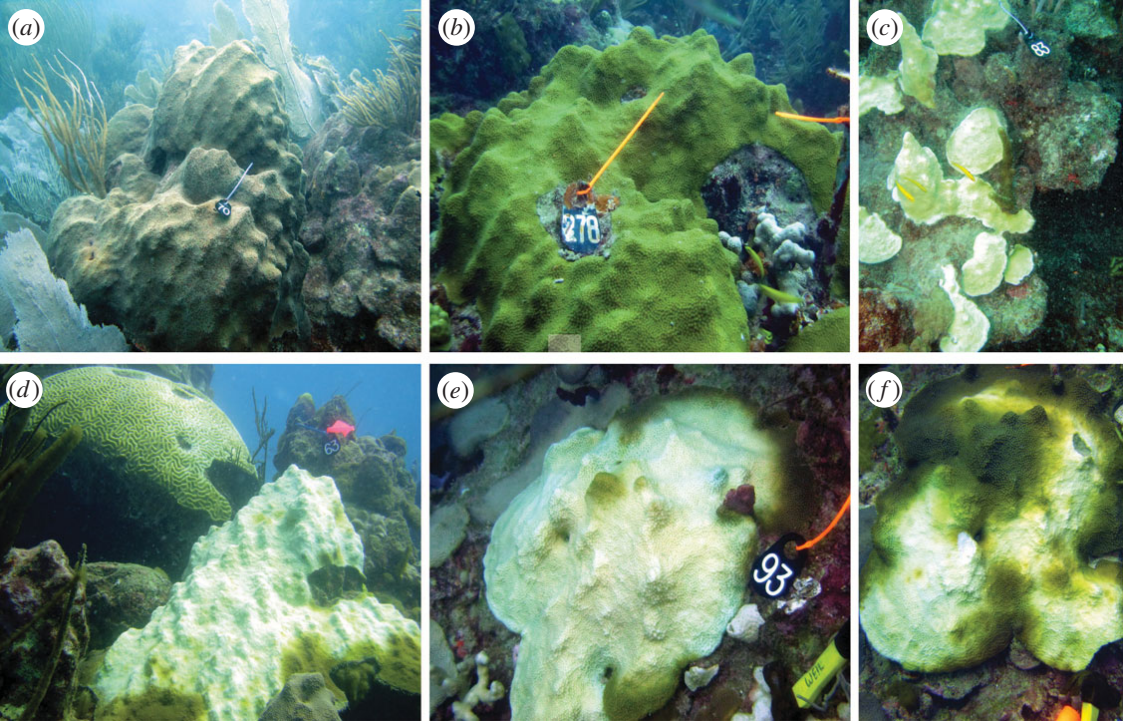
Between September and December 2010, a bleaching event affected 40% of the colonies of the reefs off La Parguera, Puerto Rico. *Orbicella faveolata* was among the species affected during this event. The images show unbleached (*a*,*b*) and bleached colonies (*c*–*e*) during the height of the bleaching in September 2010. By December 2010, some colonies ((*f*)—same colony shown in (*e*)) showed signs of recovery. Full recovery was observed in early 2011. The height of the black tags is 9 cm.

From November 2010 to October 2011, four *O. faveolata* colonies (two that appeared bleached and two with no signs of bleaching) from El Turromote reef (17°56.097^′^ N; 67°01.130^′^ W), off La Parguera (southwest Puerto Rico), were tagged and monitored over the following 11 months, ending in October 2011 (figure 2). Bleached colonies recovered normal coloration by March 2011 and remained healthy in appearance through October 2011. Unbleached colonies did not show any obvious signs of colour or pigmentation loss during the same period of time.

Coral fragments (approx. 2 cm^2^) were collected from all tagged colonies on three occasions: during bleaching (November 2010), after recovery of symbionts (four months—March 2011) and approximately a year after the first collection (11 months—October 2011). Samples were excised from the top of the colonies with a hammer and chisel, placed in sterile plastic bags and transported in seawater, at the collection site temperature, to the laboratory at the Department of Marine Sciences—University of Puerto Rico Mayagüez. Fragments were immediately flash frozen in liquid nitrogen, transported to Cornell University in dry ice and stored at −80°C until further analysis.

### 3.2 RNA extractions, cDNA library preparation and sequencing

Each individual *O. faveolata*fragment was ground in liquid nitrogen using a mortar and pestle, and the resulting powder was placed in a 2.0 ml microcentrifuge tube. Total RNA was extracted using a modified Trizol/Qiagen RNeasy protocol as in Burge *et al.* [42]. After the ethanol precipitation step, following the manufacturer's instructions (Invitrogen, Life Technologies Corporation, Grand Island, NY, USA), RNA was cleaned from aqueous solution using an RNeasy column (Qiagen, Valencia, CA, USA). DNA was removed from the extracted solution using the Turbo DNA-free treatment according to the manufacturer's instructions (Ambion, Life Technologies Corporation). Removal of DNA was confirmed by using RNA (1 μl) as template in a quantitative PCR targeting 18S ribosomal DNA as previously described [43]. RNA concentrations were quantified using the NanoDrop ND-1000 (NanoDrop Technologies, Wilmington, DE, USA).

RNA quality was assessed using a BioAnalyzer 2100 (Agilent Technologies, Santa Clara, CA, USA) at the Cornell University Biotechnology Resource Center in all 12 extracted RNA samples. All samples showed quality values with RNA integrity numbers between 9.2 and 9.8 as determined with the BioAnalyzer. Libraries were prepared using the Illumina TruSeq RNA Sample Preparation kit with poly-A selection, according to the manufacturer's protocol (including bar-coding for multiplexing) and sent to the Cornell CLC Life Sciences facility for Illumina (Hi-Seq) 100 bp paired-end sequencing. Samples were multiplexed and sequenced in a total of three lanes (eight libraries per lane, four for this manuscript and four for other projects).

### 3.3 Transcriptome assembly

After removing adapters and low-quality reads (Trimmomatic [44]), the resulting reads from all sequenced libraries were combined and used to assemble a *de novo* metatranscriptome using the package Trinity [45]. This metatranscriptome included genes from the coral host, the algal symbiont (i.e. ‘*Symbiodinium* spp.’) and ‘other-eukaryotes’ (e.g. fungi, ciliates, endolithic algae, etc.) associated with *O. faveolata*. The poly-A selection in the library preparation reduces prokaryotic sequences, thus herein we primarily discuss the eukaryotic holobiont (i.e. coral host, ‘*Symbiodinium* spp.’ and ‘other-eukaryotes’).

To characterize the coral host transcriptome and elucidate the impacts of bleaching to the coral innate immune system, the metatranscriptome was filtered with genome and transcriptome data from different species or types of *Symbiodinium* and the *O. faveolata* genome. *Symbiodinium* data included the draft genome of *Symbiodinium minutum* (type B1, strain Mf1.05b—21 899 genes, 603 716 798 bp [46]), genomic sequences from cultured *Symbiodinium* types *S. fitti* (type A3; 97 259 contigs—21 653 717 bp) and type C1 (82 331 contigs—44 078 667 bp. *Symbiodinium fitti* and C1 data provided by Todd C. LaJeunesse, The Pennsylvania State University), and transcriptome data from *S. microadriaticum* (type A, KB8 strain—72 152 contigs—61 869 232 bp from the host *Cassiopeia* spp.) and *S. minutum* (type B1, strain Mf1.05b; 76 284 contigs—45 263 394 bp from the host *O. faveolata* [47]). *Symbiodinium* sequences were combined to create a single *Symbiodinium* reference data file (349 925 contigs—776 581 808 bp; electronic supplementary material, S1) that was aligned against the metatranscriptome using BLAT [48] with 90% identity and e-value<0.000001 to filter spurious hits. Identities of the hits were filtered and duplicates removed, sequences were then retrieved from the metatranscriptome using the tool cdbfasta/cdbyank (http://sourceforge.net/projects/cdbfasta/), resulting in the ‘*Symbiodinium* spp.’ transcriptome. The metatranscriptome without the *Symbiodinium*-only genes was aligned (using BLAT as above) against the host genome (approx. 700 000 000 bp from non-symbiotic gametes) to acquire the ‘*O. faveolata’* transcriptome and the ‘other-eukaryotes’ transcriptome. This genome is available on the *O. faveolata* Genome Consortium website: http://montastraea.psu.edu/.

### 3.4 Gene expression analysis and gene ontology

Reads from each of the samples (*n*=12) were aligned against each of the transcriptomes to determine the expression levels within each of the components of the holobiont. Estimates of genes/contigs abundances for each sample and comparative gene expression analyses across samples and colony conditions through time were performed using Tophat, Cufflink and CummeRbund [49]. Changes in expression of genes involved in immunity or immune-related processes (e.g. immunity, signalling, response to stimulus) were further explored. We use the following designations to discern different conditions and time points in our sampling: bleached refers to corals that appeared white after losing their associated *Symbiodinium* cells in November 2010, during the height of the bleaching event. Even though these colonies regained their algal symbionts in March 2011, they are still referred to as bleached colonies or previously bleached colonies through the subsequent collection periods (March 2011 and October 2011). Corals that kept their pigmentation and algal cell populations in their tissues are referred to as unbleached.

Gene ontology annotations were initially determined using BLAST [50] for the metatranscriptome contigs/genes, and further explored with Protein Analysis Through Evolutionary Relationships [51] and Blast2GO [52] for genes showing significant gene expression differences (corrected *p*-values greater than 0.05). The metatranscriptome was blasted against the Swiss-Prot database. In Blast2Go, the annotations were obtained from the NCBI's nucleotide database, InterPro, GO, Enzyme Codes and KEGG. Enrichment tests among the differentially expressed genes were performed for the biological processes using the Fisher's exact test on Database for Annotation, Visualization and Integrated Discovery—DAVID v. 6.7 [53]. All biological processes except locomotion and biological regulation show *p*-values smaller than 0.05, suggesting that the processes are significantly enriched. Pathways involving genes with significant differences were obtained using PathVisio [54] from WikiPathways [55] and the Pathway Interaction Database [56].

### 3.5 *Symbiodinium* spp. type identity

The identity of the associated *Symbiodinium* types in each sample was determined with BLAST [50]. Reads from each of the samples were aligned against sequences of the internal transcribed spacer 2 (ITS2) of *Symbiodinium* types known to inhabit *O. faveolata* (*S. fitti*, D1a, *S. minutum*, C3, C3d, C3e, C7, C12). Alignments with 100% match were use as the correct identity. The symbiont identity of additional samples of the same colonies but collected in other months (September and December 2010 and August 2011) was determined using denaturing gradient gel electrophoresis of the ITS2 region [57–62].

## 4. Results

After trimming and quality filtering, a total of 387 512 512 pair-end reads (32 292 709±4 147 919 reads/library) were retained, with an average length of 75 bp. Sequences were deposited in the National Center for Biotechnology Information Short Read Archive under the SRP022773 accession number. The metatranscriptome was assembled with all retained reads, resulting in 442 294 contigs (401 528 469 bp, N50=1551) with an average length of 908 bp (table 1 and electronic supplementary material, S2). Filtering the metatranscriptome with genomic and transcriptomic data allowed for the separation of the contigs from the metatranscriptome into three transcriptomes, one for each of the components of the holobiont; ‘*O. faveolata*’, ‘*Symbiodinium* spp.’ and ‘other-eukaryotes’. The ‘*O. faveolata*’ transcriptome had 178 943 contigs, the ‘*Symbiodinium* spp. transcriptome’ had 130 217 contigs and the ‘other-eukaryotes’ transcriptome had 202 236 contigs (table 1 and electronic supplementary material, S3, S4 and S5, respectively). Some conserved genes may have been classified as being part of more than one transcriptome, resulting in overlap across the three transcriptomes. As expected, the coral was highly represented within samples with an average of 77.5% of the raw reads aligning with the ‘*O. faveolata*’ transcriptome (table 2). The other two components of the holobiont aligned with 7.6% (‘*Symbiodinium* spp.’ transcriptome) and 8.5% (‘other-eukaryotes’ transcriptome) of the raw reads.

**Table 1.** Statistics of the sequencing data for the *O. faveolata* holobiont (metatranscriptome) and for each of its components (‘*O. faveolata*’, ‘*Symbiodinium* spp.’ and ‘other-eukaryotes’). The transcriptomes can be found in the corresponding electronic supplementary material files.

	metatranscriptome (electronic supplementary material, S2)	
	retained reads	387 512 512 (75 bp average length)
	no. contigs	442 294 (401 528 469 bp)
	average length	908 bp (min—201 bp; max—38 110 bp)
	N50	1551
	no. annotated contigs	108 409
	*O. faveolata* (electronic supplementary material, S3)	
	no. contigs	178 943 (196 757 464 bp)
	average length	1100 bp (min—201 bp; max—38 110 bp)
	N50	2218
	no. annotated contigs	41 584
	*Symbiodinium* spp. (electronic supplementary material, S4)	
	no. contigs	130 217 (172 005 919 bp)
	average length	1321 bp (min—201 bp; max—15 175 bp)
	N50	1844
	no. annotated contigs	22 157
	other-eukaryotes (electronic supplementary material, S5)	
	no. contigs	202 236 (136 560 496 bp)
	average length	675 bp (min—201 bp; max—13 602 bp)
	N50	1100
	no. annotated contigs	45 583

**Table 2.** Total number of reads used in the assembly of the *O. faveolata* metatranscriptome and those aligned against each of the holobiont components (‘*O. faveolata*’, ‘*Symbiodinium* spp.’ and ‘other-eukaryotes’). Percentages are of the total of the raw reads for each row.

	raw reads		aligned reads							
month	colony condition and no.	total	*O. faveolata*	%	*Symbiodinium* spp.	%	other-eukaryotes	%	total	%
bleaching										
November 2010	unbleached 1	25 263 324	18 397 266	72.8	3 458 682	13.7	1 760 588	7.0	23 616 536	93.5
	unbleached 2	22 167 068	17 899 877	80.7	2 400 183	10.8	838 853	3.8	21 138 913	95.4
	bleached 1	17 619 525	15 869 696	90.1	292 570	1.7	143 158	0.8	16 305 424	92.5
	bleached 2	17 321 791	15 740 825	90.9	1 098 670	6.3	161 552	0.9	15 902 377	91.8
post-bleaching										
March 2011	unbleached 1	45 411 481	35 655 303	78.5	5 944 196	13.1	1 160 146	2.6	42 759 645	94.2
	unbleached 2	21 592 782	15 583 474	72.2	1 855 259	8.6	3 018 662	14.0	204 57395	94.7
	bleached 1	33 446 863	25 284 275	75.6	1 377 763	4.1	4 268 640	12.8	30 930 678	92.5
	bleached 2	64 535 106	50 983 253	79.0	4 804 314	7.4	5 339 275	8.3	56 322 528	87.3
October 2011	unbleached 1	31 158 747	22 055 972	70.8	949 427	3.0	6 541 857	21.0	29 547 256	94.8
	unbleached 2	50 476 948	40 373 490	80.0	6 881 860	13.6	1 144 076	2.3	48 399 426	95.9
	bleached 1	30 226 535	21 934 584	72.6	1 376 168	4.6	4 297 944	14.2	26 232 528	86.8
	bleached 2	28 292 342	18 843 835	66.6	1 207 627	4.3	4 005 816	14.2	18 843 835	66.6
	average	32 292 709	24 885 154	77.5	2 894 993	7.6	2 606 796	8.5	29 204 712	93.6

### 4.1 Annotations and gene ontology

Annotations, using the Swiss-Prot database, were possible for 108 409 (approx. 24.5%, table 1 and electronic supplementary material, S6) of the 442 294 contigs in the metatranscriptome. Of these annotated genes, 41 584 corresponded to ‘*O. faveolata*’, 22 157 to ‘*Symbiodinium* spp.’ and 45 583 to ‘other-eukaryotes’ transcriptomes (table 1). The most informative partition was the ‘*O. faveolata*’ transcriptome, as it represented all the contigs/genes found in a single species (i.e. *O. faveolata*). The other two transcriptomes were made up of transcripts from multiple taxa, from several *Symbiodinium* species in the ‘*Symbiodinium* spp.’ transcriptome, and likely numerous protistan/fungal lineages in the ‘other-eukaryotes’ transcriptome. Alignments with *Symbiodinium* sequences of the ITS2, revealed the presence of at least four different types of *Symbiodinium* (*S. fitti*, D1a, C7 and *S. minutum*) in the dataset.

### 4.2 The *Orbicella faveolata* only transcriptome

**Figure 3.**
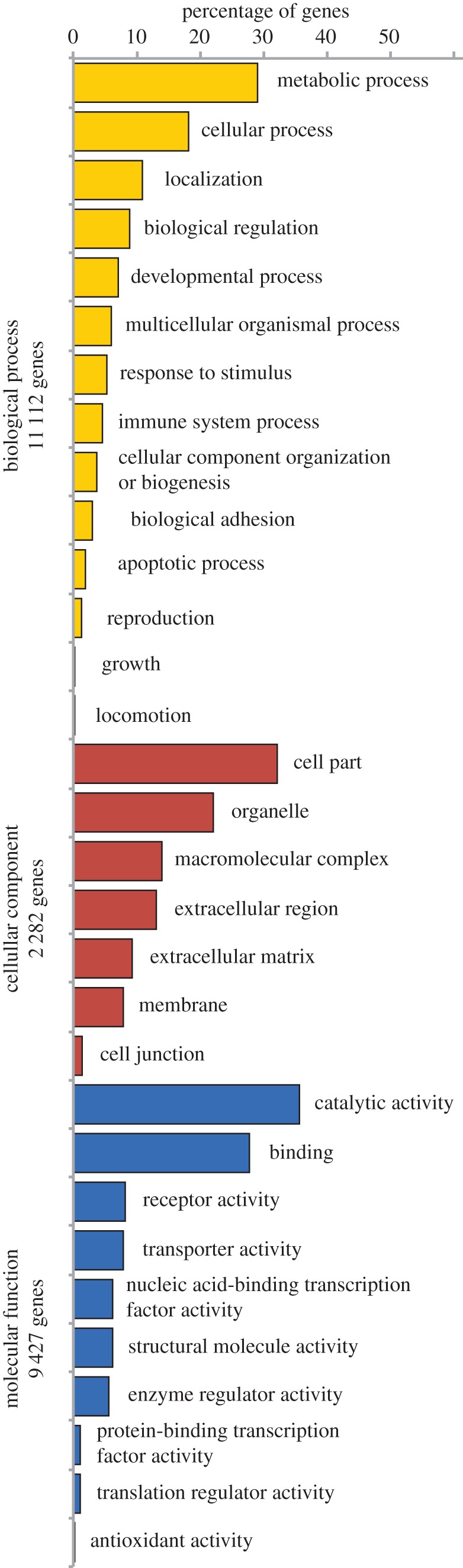
Percentage of genes involved in several biological processes, cellular component and molecular functions found in the ‘*O. faveolata*’ transcriptome assembled from bleached and unbleached colonies collected during and after the 2010 natural coral bleaching event in La Parguera, Puerto Rico. Categories determined after gene ontology analysis.

Gene ontology revealed that the annotated genes found in the ‘*O. faveolata*’ transcriptome belong to 14 different biological processes, 10 molecular functions and seven cellular components (figure 3). Among the biological processes, metabolic processes (GO:0008152—5278 genes), cellular processes (G0:0009987—3300 genes) and localization (GO:0051179—1973 genes) represented in combination 58.2% of all the hits. Immune-related processes included response to stimulus (GO:0050896—960 genes), immune system processes (GO:0002376—834 genes), biological adhesion (GO:0022610—542 genes) and apoptotic processes (GO:0006915—339 genes). In terms of molecular function, the categories with higher hits were: catalytic activity (GO:0003824—3361 genes) and binding (GO:0005488—2620 genes). The most represented cellular components were cell part (GO:0044464—734 genes) and organelle components (GO:0043226—504 genes; figure 3).

### 4.3 Effects of coral bleaching and the response of the coral holobiont

**Figure 4.**
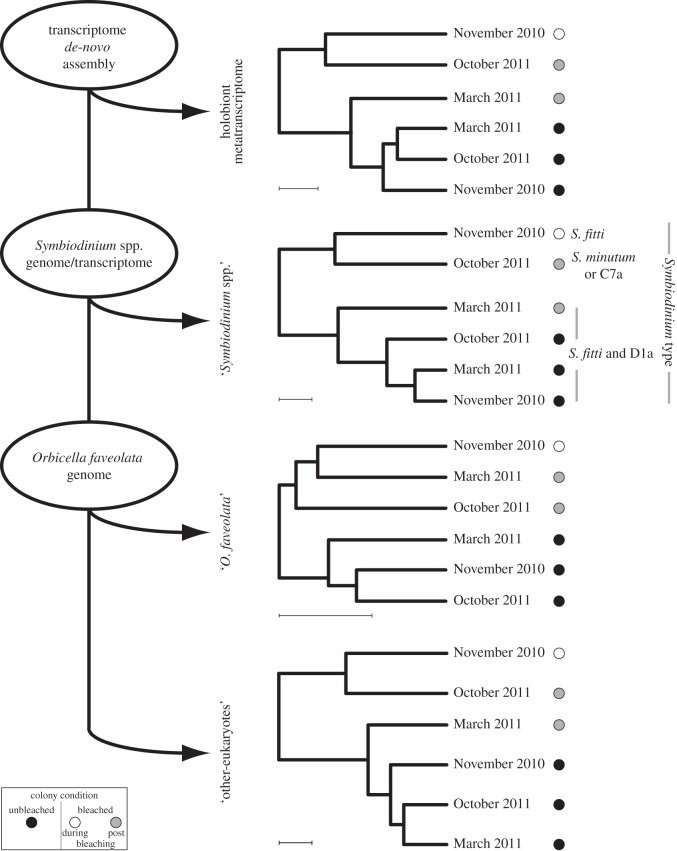
Schematic of the RNA-seq analyses on colonies of the Caribbean coral *O. faveolata* collected during and after the 2010 coral bleaching in La Parguera, Puerto Rico. RNA-seq reads from all samples (*n*=12) were grouped together to built a reference transcriptome and then filtered with genomic and transcriptomic data from several *Symbiodinium* types, as well as with the *O. faveolata* genome, to generate expression profiles for the holobiont (i.e. metatranscriptome), ‘*Symbiodinium* spp.’, ‘*O. faveolata*’ and ‘other-eukaryotes’. ‘*Orbicella faveolata*’ profile shows a different pattern to that seen in the other profiles due to the clustering of the bleaching and unbleached colonies in separate groups. Letters next to the ‘*Symbiodinium* spp.’ profile depict the *Symbiodinium* type found in the sequenced colonies. Identities of *Symbiodinium* types were obtained with a BLAST alignment performed against ITS2 sequence data from types known to associate with the coral *O. faveolata*.

Analyses of gene expression of the holobiont (i.e. metatranscriptome) resulted in 6562 unique genes with significant (*p*-values<0.05 after false rate discovery correction) differences in expression across all samples. Gene expression levels across time (November 2010 and March and October 2011) and colony condition (bleached versus unbleached) revealed most of the differences were between bleached and unbleached colonies during the bleaching event (November 2010; figure 4). Unexpectedly, colonies that bleached showed similar whole expression profiles when they were bleached and nearly a year later, even though these colonies returned to normal coloration and symbiont cell density by March 2011 (similar levels of *Symbiodinium* density to those of unbleached colonies).

Further analyses of the ‘*Symbiodinium* spp.’ and ‘other-eukaryotes’ expression profiles revealed similar if not the same patterns as those seen in the metatranscriptome analysis (figure 4). Molecular identification of the symbiont present in each sample showed differences between bleached and unbleached colonies. Bleached colonies showed changes in the dominant symbiotic species, contrary to non-bleached colonies where the association was stable during the sampling period. In bleached colonies, two changes in dominance occurred: from *S. fitti* (type A3) during bleaching to *S. fitti* and D1a in March 2011 and to *Symbiodinium* C7a and *S. minutum* (B1)/B2 in August 2011. By contrast, in unbleached colonies the symbiosis remained stable, forming associations with *S. fitti* and the thermally tolerant D1a through the year (figure 4).

**Figure 5.**
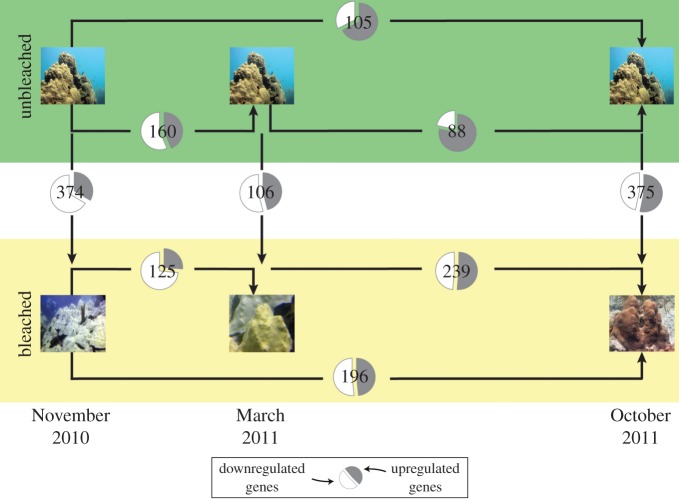
Number of regulated genes with significant differences in expression (number inside the pie charts) between bleached and unbleached colonies and across sampling times. Unbleached colonies have less regulated genes than bleached colonies. In the pie chart, the proportion of genes upregulated in the colonies is represented in grey and the downregulated proportion is in white.

Whole transcriptome expression analyses of the coral host between time and colony condition revealed a slightly different profile to those from the metatranscriptome and the ‘*Symbiodinium* spp.’ and ‘other-eukaryotes’ transcriptomes. In the coral *O. faveolata*, expression profiles formed two clusters, one comprising samples from the bleached colonies and the other with unbleached colonies (figure 4). A total of 1368 unique genes showed significantly different expression levels. The number of genes with changes in expression levels across collection months (November 2010, March 2011 and October 2011) and colony condition (i.e. bleached versus unbleached) was variable (figure 5), with more differences between bleached and unbleached colonies during the height of the event in November 2010 (374 genes) and in October 2011 (375 genes) than in March 2011 (106 genes). Additionally, bleached colonies showed more genes with significant differences (125–239 genes) during the sampling period than unbleached colonies (88–160 genes; figure 5). Differences in the number of expressed genes over the surveyed year appeared to follow a seasonal pattern but bleached colonies deviated from this pattern after March 2011. Expression levels of bleached colonies in October 2011 were similar to those detected during the bleaching event in November 2010. Changes in whole transcriptome expression levels suggest that gene expression changes in the coral host appear to be persistent at least 1 year after bleaching.

**Figure 6.**
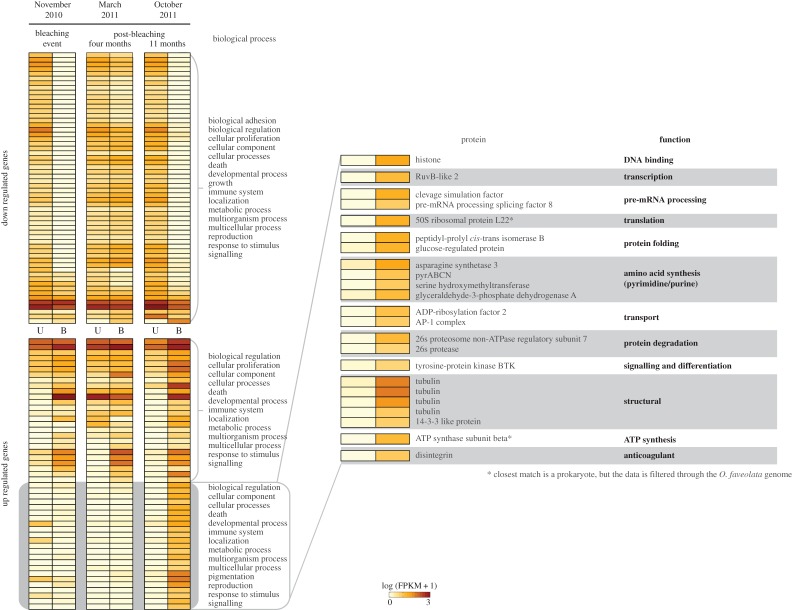
Patterns of expression of the coral *O. faveolata*seen 11 months post-bleaching (October 2011) were similar to those seen during the bleaching event (November 2010) even though these annotated genes appeared to be expressed at similar levels between bleached and unbleached colonies only four months post-bleaching (March 2011). Annotated genes that were upregulated (upper panel) in bleached colonies in October 2011 were also upregulated in November 2010. A similar situation is seen with the downregulated genes from October 2011 (lower panel). The exception were 24 genes that appear upregulated in October 2011 (enlarged panel), all these genes are involved in transcription, translation of proteins as well as transport and degradation, suggesting bleached coral colonies might be trying to compensate for the lack of expression in other genes.

Of the 1368 genes showing significant differences in expression levels across collection times (November 2010 and March and October 2011) and colony condition (bleached versus unbleached), 729 (approx. 53%; electronic supplementary material, S7) were annotated. These genes are involved in several gene ontology categories, including immune-related (signalling GO:0023052, responses to stimulus GO:0050896 and immune system processes GO:0002376), metabolism (cell component organization or biogenesis GO:0071840, metabolic processes GO:0008152 and cellular processes GO:0009987) and reproduction (reproduction GO:0000003 and cell proliferation GO:0008283). Annotated genes showed similar patterns of expression between bleached and unbleached colonies during November 2010 and October 2011, with the pattern being different in March 2011. For example, upregulated genes in bleached colonies in November 2010 were also upregulated in October 2011, but their expression levels in March 2011 were similar to those in unbleached colonies (figure 6). However, another pattern was apparent in bleached colonies, where 24 genes (out of the 729 annotated genes) appeared to shift their expression levels from downregulated during bleaching (November 2010) to upregulated 11 months after bleaching (October 2011; figure 6), when bleached colonies appear to have recovered their *Symbiodinium* cell densities. These genes are involved in several pathways with various functions, including DNA binding, transcription, RNA processing, protein folding, protein transport, protein degradation, signalling and structural components (figure 6).

### 4.4 Effects of bleaching on genes of the coral host immune system

**Figure 7.**
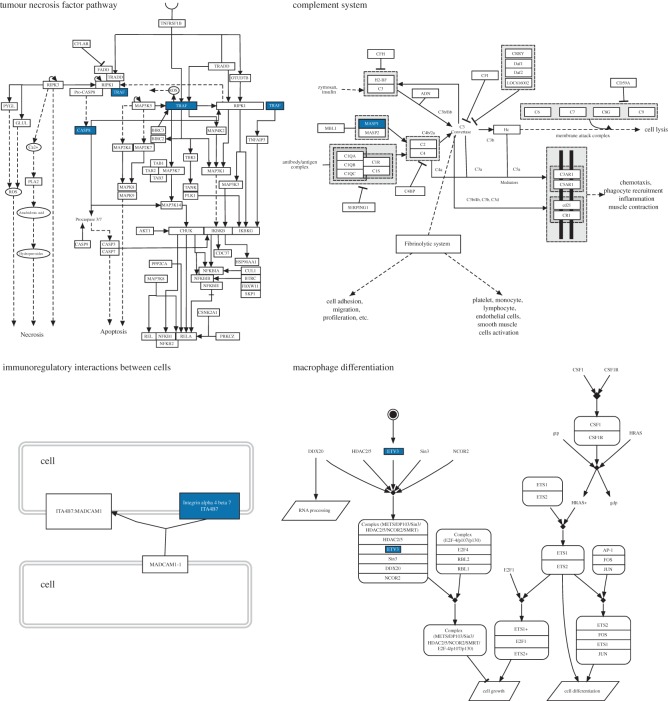
Simplified versions of four important immune-related pathways affected in bleached colonies during the 2010 bleaching event in La Parguera, Puerto Rico. The genes highlighted in blue correspond to some of those with significant changes in expression while the colonies were bleached (November 2010), during the recovery phase (March 2011) and/or a year after the event (October 2011). The expression profiles of these genes can be found in figure 8. Pathways were obtained from WikiPathways [55] and the Pathway Interaction Database [56] and edited in PathVisio [54].

**Figure 8.**
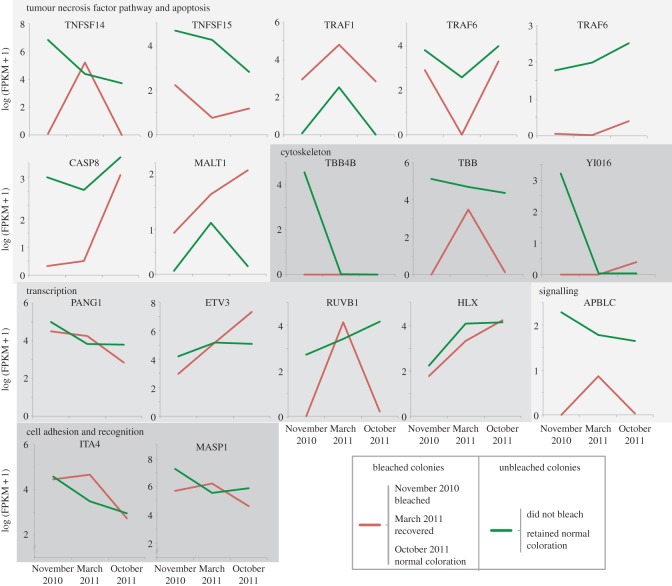
Changes in gene expression of 17 immune-related genes of *O. faveolata*, during (November 2010) and after (March and October 2011) the 2010 bleaching event in La Parguera, Puerto Rico, for both bleached (red) and unbleached (green) colonies. The genes are grouped in five functional groups, tumour necrosis factor pathway and apoptosis, cytoskeleton, transcription, signalling and cell adhesion and recognition.

Under the immune system processes GO category (GO:0002376), 17 genes presented significant differences between collections months (November 2010 and March and October 2011) and colony condition (bleached versus unbleached). These genes can be clustered into five functional groups: tumour necrosis factor pathway and apoptosis, cytoskeleton, transcription, signalling and cell adhesion, and recognition (figures 7 and 8). In the first group, there are two tumour necrosis factor ligands (TNFSF14 and 15), and three receptor-associated factors (TRAF1 and 2, TRAF6), a caspase (CASP8) and a mucosal-associated lymphoid tissue lymphoma translocation protein (MALT1). The cytoskeleton group included three tubulin beta proteins (TBB4B, TBB and YI016). The transcription group has four genes: protein pangolin (PANG1), ETS translocation variant 3 (ETV3), RuvB-like 1 (RUVB1) and a homeobox-like protein (HLX). The signalling group has one protein, the beta adaptin-like protein (APBLC). The cell adhesion and recognition group has an integrin alpha 4 (ITA4) and a mannan-binding lectin serine protease (MASP1).

Expression levels of the 17 aforementioned immune genes were downregulated in bleached colonies compared to unbleached colonies in November 2010 (figure 8). Only two genes (TRAF1 and MALT1) showed higher expression values in bleached than in unbleached colonies. At the end of the survey (October 2011), bleached colonies showed lower expression values in 10 of these genes (TNFSF14/15, TRAF6, CASP8, MASP1, TBB, APBLC, PANG and RUVB1), similar expression levels in four genes (ITA4, TBB4b, YI016 and HLX) and higher expression levels in three genes (TRAF1, MATL1 and ETV3), compared with expression levels in unbleached colonies. The expression of TRAF1, MATL1 and ETV3 do not follow the patterns seen in the other immune-related genes. TRAF1 deviated from the expression levels seen in the other tumour necrosis factor pathway genes and is upregulated in the bleached colonies. MATL1 was also upregulated in bleached colonies and had high expression levels in October 2011. Finally, ETV3 through the survey appeared as a low expressed gene in bleached colonies, compared with unbleached colonies, but in the last month (October 2011) its expression increased to higher levels than those seen in the unbleached colonies (figure 8).

## 5. Discussion

High-throughput sequencing is becoming a very common tool to answer important questions about the impacts of climate change on scleractinian corals [31,63–66]. Our metatranscriptome analysis of the important reef-building coral *O. faveolata* is enhanced by the draft genome of the same host species. Although limited in sample numbers per time and condition, this study incorporates data from a natural coral bleaching event and subsequent recovery phase in the same coral colonies, resulting in a more comprehensive analysis of the processes involved in bleaching and recovery. The results from this study highlight the lasting effect coral bleaching has on key biological, physiological and immune pathways.

### 5.1 The composition of the coral-symbiotic community masks the response of the coral host

The clustering of gene expression profiles of the holobiont shows grouping of only the bleached colonies from November 2010 and October 2011. Interestingly, bleached colonies from March 2011 grouped with all the unbleached colonies. Upon closer examination of the individual components of the holobiont, the profiles of ‘*Symbiodinum* spp.’ and ‘other-eukaryotes’ transcriptomes have the same pattern as each other and the metatranscriptome. The coral host, however, has a different pattern of expression profiles with all the bleached samples clustering together regardless of month of collection.

Bleached coral colonies show shifts in the dominant *Symbiodinium* types. The gene expression profiles of the ‘*Symbiodinium* spp.’ transcriptomes, therefore, appear to be driven by the identity and the genetic composition of the *Symbiodinium* type inhabiting the colony at that time. All unbleached colonies maintained the same *Symbiodinium* types (*S. fitti* and D1a) and their profiles were similar to each other. On the other hand, bleached colonies showed shifts in *Symbiodinium.*During March 2011, bleached colonies acquired D1a in addition to *S. fitti* they already had and their gene expression profiles resembled that of the unbleached colonies that harboured the same types throughout. D1a has been proposed as a stress tolerant symbiont and may have helped these colonies recover [67,68]. The ‘other-eukaryotes’ portion of the holobiont may also have similar community shifts, but more resolution in the identity and function of these communities is needed.

The response of the holobiont reflected the expression levels of the less represented portions in our RNA libraries, leading to the masking of the expression of the coral host. Reads aligning to the ‘*Symbiodinium* spp.’ and ‘other-eukaryotes’ transcriptomes represented a low percentage of the total number of reads obtained during sequencing. This observation suggests that the overall condition of the colony is a result of the physiological tolerance of each of the elements of the holobiont, but can also be a reflection of the different genetic composition of each portion across time [69–73].

### 5.2 Bleaching affects several biological processes in the coral host, even a year after the event

Coral bleaching not only affects the coral–algae relationship but also acts on several aspects of the physiology and ecology of corals [12,21–26]. Here, the regulation in the levels of gene expression in bleached colonies provides evidence of some of the affected processes. For example, reduction in epithelial tissue thickness [22] and protein synthesis [24] can be related to the downregulation of transcription, RNA processing and translation and protein synthesis and degradation during bleaching (November 2010; figure 6). Most of these processes are still affected (i.e. downregulated in bleached compared to unbleached colonies) a year after coral bleaching. However, a group of genes involved in protein synthesis and transport were upregulated in the bleached colonies 1 year after bleaching, perhaps in an effort to overcompensate the observed downregulation observed during the bleaching event.

### 5.3 Immune-related genes are affected by bleaching

Analyses of specific genes indicate that immune-related pathways such as apoptosis and the complement system are suppressed during bleaching and a year later in bleached colonies. Apoptosis plays a role in life-history processes, such as metamorphosis [74] and symbiosis [75], and it has been suggested to play a role in the defence of corals against pathogens [76,77]. Components of the tumour necrosis factor pathway and of capsase-8 were suppressed in *O. faveolata* bleached colonies. It is likely that the initial suppression of apoptosis in the bleached colonies (i.e. November 2010) is related to the mechanisms controlling bleaching. When apoptosis is blocked, bleaching can be reduced [78,79]. Although initially a mechanism to mitigate bleaching, continued downregulation of apoptosis 11 months after bleaching can have an immunosuppressive effect [76,77]. Bleached *O. faveolata* colonies are known to have higher disease prevalence than unbleached colonies during and after bleaching [11].

The complement system tags or selects foreign molecules for destruction [80,81]. A key component of this system is the mannan-binding lectin serine protease 1 (MASP1), which appears to be the exclusive activation factor of the pathway and produces large amounts (60%) of C2a, a compound responsible of C3 convertase formation [82]. MASP1 in bleached *O. faveolata* colonies was downregulated early during the recovery phase (March 2011), compared to during bleaching (November 2010) and 11 months after bleaching (October 2011). The pattern however was the opposite in unbleached colonies. Differences in expression of MASP1 between bleached and unbleached colonies suggest that the complement system is inactive, or less active, in bleached colonies. A less active component system indicates the lack, reduction or suppression of the immune system. In addition to apoptosis and the complement system, the cytoskeleton and translation are affected and genes such as RuvB [83,84] are downregulated in bleached colonies.

Environmental stressors have been linked to immunosuppression in other invertebrates [85]. The increase in new diseases and disease prevalence after bleaching [25,26] can be the result of the host immunosuppression during and after bleaching. The immune system is a well-regulated network of processes and pathways [86] that can interact with other cellular pathways. Upregulation of some genes involved in protein synthesis (e.g. peptidyl-prolyl *cis*-trans isomerase B, asparagine synthetase 3) and transport (e.g. ADP-ribosylation factor 2) and structural proteins (e.g. tubulin) a year after bleaching might be an attempt by the bleached colonies to compensate for their immunosuppression.

### 5.4 Concluding remarks

This study provides evidence that the coral holobiont and the coral host have different responses in terms of gene expression, during bleaching and through the recovery process. Here, we present evidence on previously unknown effects of bleaching; (i) Results of the metatranscriptome analysis indicate that each portion of the holobiont (i.e. ‘*O. faveolata*’, ‘*Symbiodinium* spp.’ and ‘other-eukaryotes’) has different responses to and recovery from bleaching; (ii) the coral host response appears to be masked by the responses of the associated organisms (i.e. ‘*Symbiodinium* spp.’ and ‘other-eukaryotes’); (iii) bleached colonies may not successfully recover from bleaching, while unaffected colonies do not experience as intense changes in gene expression; and (iv) the effects of bleaching on the host immune system extend beyond recovery of the *Symbiodinium* population and appear to result in immune suppression. These results support the hypothesis that coral bleaching affects the expression of innate immune genes of corals, and these effects can last up to a year after the event.

Analyses on thermal resistance of corals suggest that some individuals might be able to overcome rising temperatures associated with climate change [31–34]. Bleaching impacts have proven erratic, and corals that in the past survived such events have been locally exterminated in the same locations after new bleaching events [87]. Results in this paper suggest that bleaching has long-term effects, but at the same time provide evidence that unbleached corals remain better prepared to fight pathogenic infections. The relation between coral bleaching and immunity in corals is complex and variable [30]. Studies emphasizing the role of coral immunity as an important aspect in coral's resistance to stressors can help improve predictions of the future of corals and coral reefs [88,89].

## Supplementary Material

Electronic supplementary material 1. Fasta file containing the Symbiodinium genomic data used to filter the Orbicella faveolata metatranscriptome. This file includes contigs from Symbiodinium types A, A3, B1 and C1. For further details on the A3 and C1 data contact Dr. Todd C. LaJeunesse at The Pennsylvania State University. Electronic supplementary material 2. Fasta file containing 442294 contigs of the assembled Orbicella faveolata metatranscriptome. Electronic supplementary material 3. Fasta file containing 178943 contigs that constitute the ‘Orbicella faveolata’ transcriptome. Electronic supplementary material 4. Fasta file containing 130217 contigs that constitute the ‘Symbiodinium spp.’ transcriptome. Electronic supplementary material 5. Fasta file containing 202236 contigs that constitute the ‘other-eukaryotes’ transcriptome. Electronic supplementary material 6. Blast results for all genes found in the Orbicella faveolata metatranscriptome. Electronic supplementary material 7. List of 1368 genes from the ‘Orbicella faveolata’ transcriptome with significant differences in expression levels. Of the 1368 genes, 729 were annotated.

## Data accessibility

All raw reads have been submitted to the National Center for Biotechnology Information Short Read Archive under the SRP022773 accession number.
