# Supplementary material for: Whole transcriptome analysis reveals changes in expression of immune-related genes during and after bleaching in a reef-building coral
Source: R Soc Open Sci. 2015 Apr 1;2(4):140214. doi: 10.1098/rsos.140214 (PMC4448857; doi:10.1098/rsos.140214)
Supplement: Electronic supplementary material 1. Fasta file containing the Symbiodinium genomic data used to filter the Orbicella faveolata metatranscriptome. This file includes contigs from Symbiodinium types A, A3, B1 and C1. For further details on the A3 and C1 data contact Dr. Todd C. LaJeunesse at The Penn [file rsos140214supp1.docx]

**Electronic Supplementary Material (ESM)**

The ESM files were not uploaded in manuscript central. As suggested by the Charlotte Wray, the files associated with this manuscript have been uploaded to:

<http://wweb.uta.edu/cos/forms/pinzon.aspx>

login: guest

password: coral123

These files will be submitted to the Dryad repository once the manuscript is accepted and to the U. of Texas at Arlington Research Commons system.

The legends are:

Electronic supplementary material 1. Fasta file containing the *Symbiodinium* genomic data used to filter the *Orbicella faveolata* metatranscriptome. This file includes contigs from *Symbiodinium* types A, A3, B1 and C1. For further details on the A3 and C1 data contact Dr. Todd C. LaJeunesse at The Pennsylvania State University.

Electronic supplementary material 2. Fasta file containing 442294 contigs of the assembled *Orbicella faveolata* metatranscriptome.

Electronic supplementary material 3. Fasta file containing 178943 contigs that constitute the ‘*Orbicella faveolata*’ transcriptome.

Electronic supplementary material 4. Fasta file containing 130217 contigs that constitute the ‘*Symbiodinium* spp.’ transcriptome.

Electronic supplementary material 5. Fasta file containing 202236 contigs that constitute the ‘other-eukaryotes’ transcriptome.

Electronic supplementary material 6. Blast results for all genes found in the *Orbicella faveolata* metatranscriptome.

Electronic supplementary material 7. List of 1368 genes from the ‘*Orbicella faveolata’* transcriptome with significant differences in expression levels. Of the 1368 genes, 729 were annotated.
